# Supplementary material for: Cryo-EM structure of cadmium-bound human ABCB6
Source: Commun Biol. 2024 May 31;7:672. doi: 10.1038/s42003-024-06377-1 (PMC11143254; doi:10.1038/s42003-024-06377-1)
Supplement: Supplementary file 2 — Supplementary Information [file 42003_2024_6377_MOESM2_ESM.pdf]

Supplementary Information

for

Cryo-EM structure of cadmium-bound human ABCB6

Seung Hun Choi[1], Sang Soo Lee[1], Hyeon You Lee[1], Subin Kim[1], Ji Won Kim[2] & Mi Sun Jin[1]*

[1]School of Life Sciences, GIST, 123 Cheomdangwagi-ro, Buk-gu, Gwangju, 61005, Republic of Korea, [2]Department of Life Sciences, POSTECH, 77 Cheongam-Ro, Nam-gu, Pohang 37673, Republic of Korea

Correspondence to: misunjin@gist.ac.kr

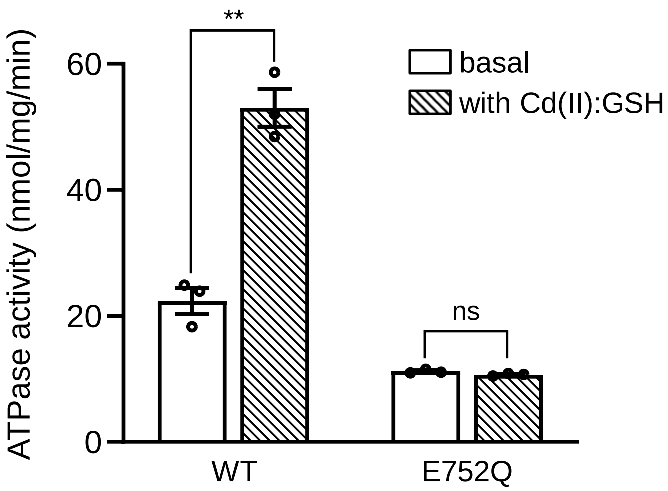

Supplementary Figure 1. ATPase activity of hABCB6^{core}-WT and the E752Q mutant. ATPase activities were measured in the presence or absence of 800 μM Cd(II) and 1 mM GSH. Mean values ± SEM were determined from triplicate measurements using the same batch of purified protein. The symbol ** represents a significant difference (*p* <0.001) between ATPase activities, with p-values calculated by a two-sided unpaired t-test and adjusted using the Welch's correction method.

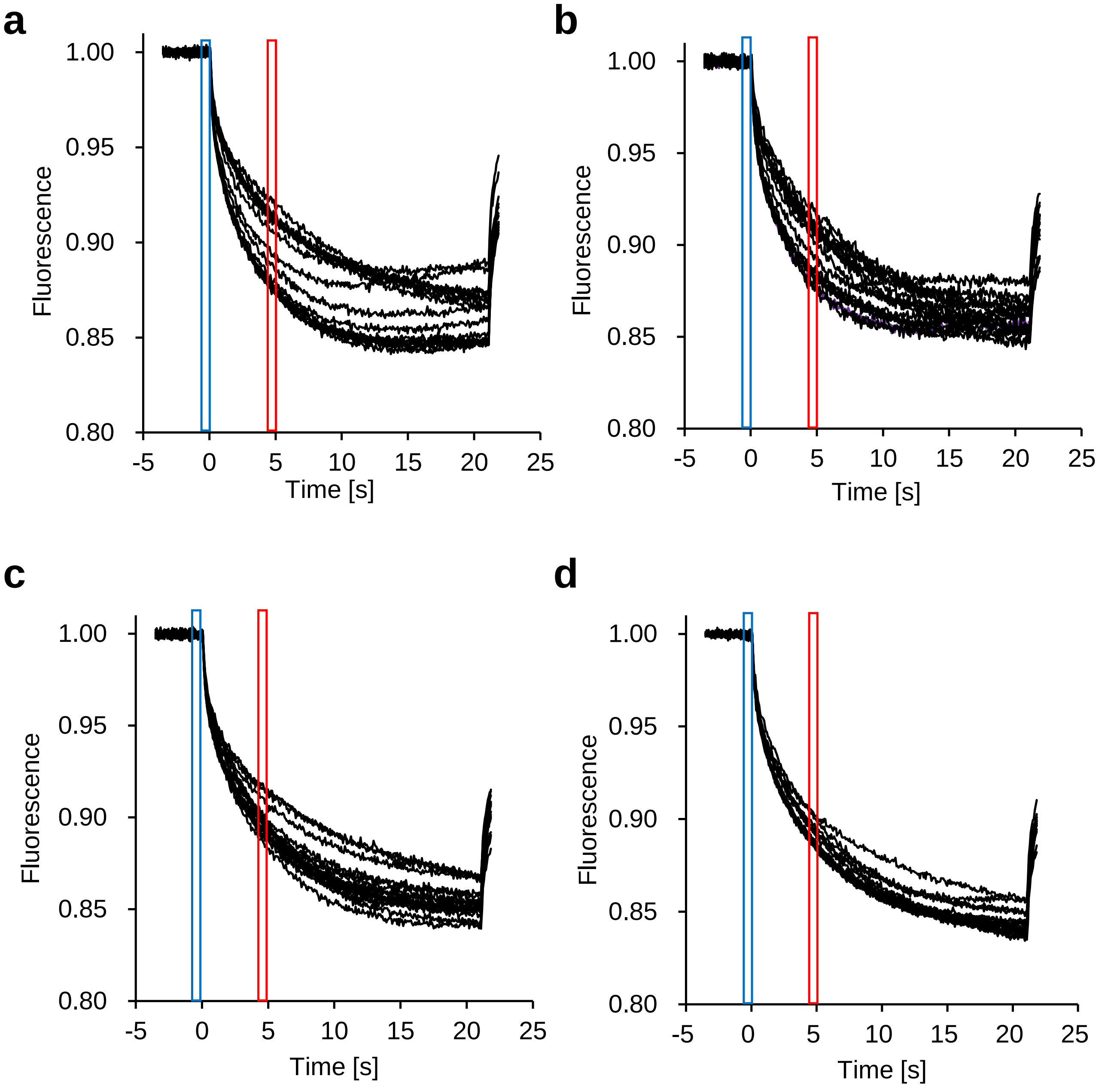

Supplementary Figure 2. Microscale thermophoresis (MST) analysis. MST raw traces were generated by analyzing 16 capillaries, each containing a constant concentration of 10 nM of fluorescently-labeled protein, and increasing concentrations of Cd(II) from a low (0 mM) to a high (62.5 mM) range in the presence of 1 μM GSH. F_{cold} (blue region) at 0 s represents the cooled state and F_{hot} (red region) at 5 s represents the heated state during thermophoresis. (a) hABCB6^{core}-WT, (b) hABCB6^{core}-E752Q, (c) hABCB6^{core}-Q501A, and (d) hABCB10^{core}-WT.

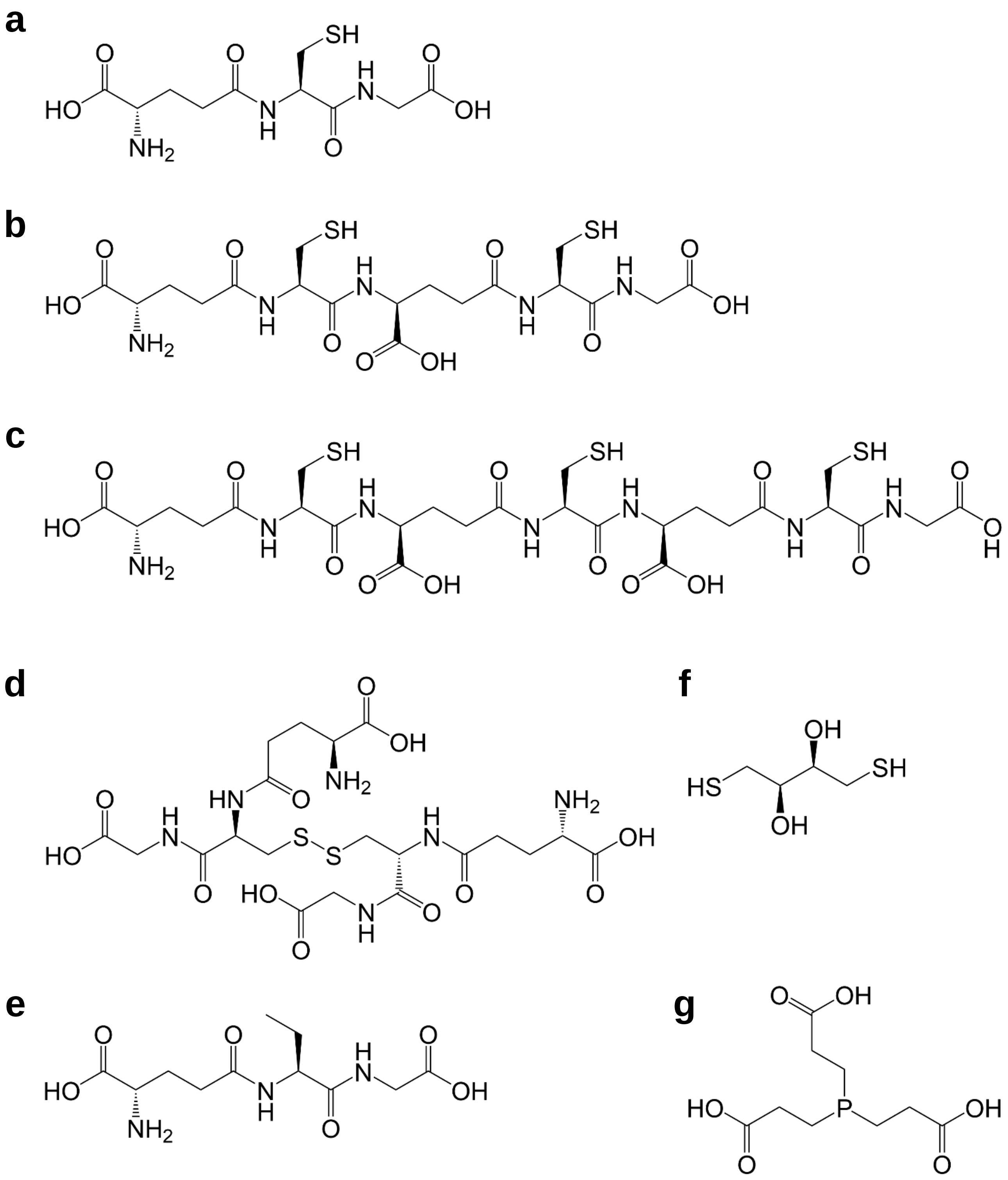

Supplementary Figure 3. Chemical structure of the various GSH derivatives and reducing agents used in this study. (a) Glutathione (GSH; γ-Glu-Cys-Gly). (b) Phytochelatin 2 (PC2; (γ-Glu-Cys)$_2$-Gly). (c) Phytochelatin 3 (PC3; (γ-Glu-Cys)$_3$-Gly). (d) Glutathione disulfide (GSSG). (e) Ophthalmic acid (OPT). (f) 1,4-Dithiothreitol (DTT). (g) Tris(2-carboxyethyl)phosphine (TCEP).

ABCB6_Cd(II):GSH-bound form

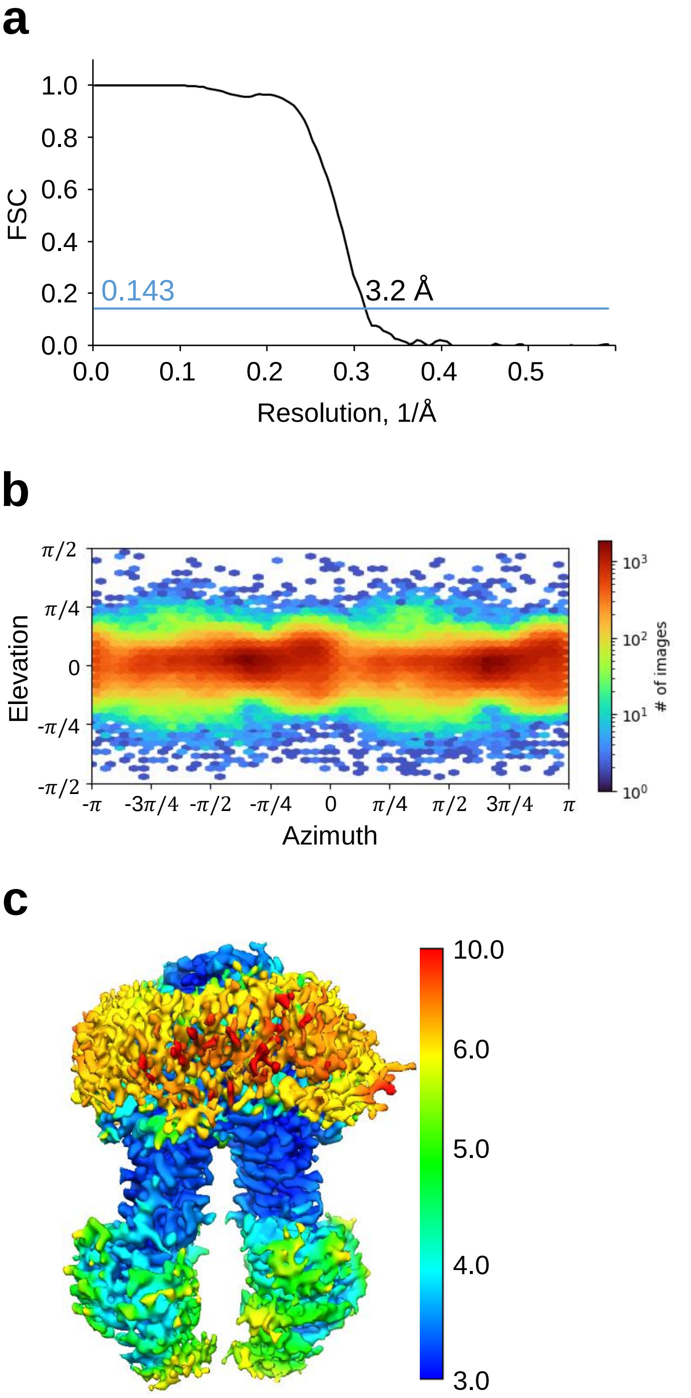

Supplementary Figure 4. Cryo-EM data and model quality analysis of Cd(II):GSH-bound hABCB6^{core}. (a) Gold-standard Fourier Shell Correlation (FSC) curve. The resolution estimation (blue line) was calculated based on an FSC cut-off of 0.143. (b) Particle distribution plot for the final 3D reconstruction. (c) Density map colored by local resolution estimation from 3 Å (blue) to 10 Å (red).

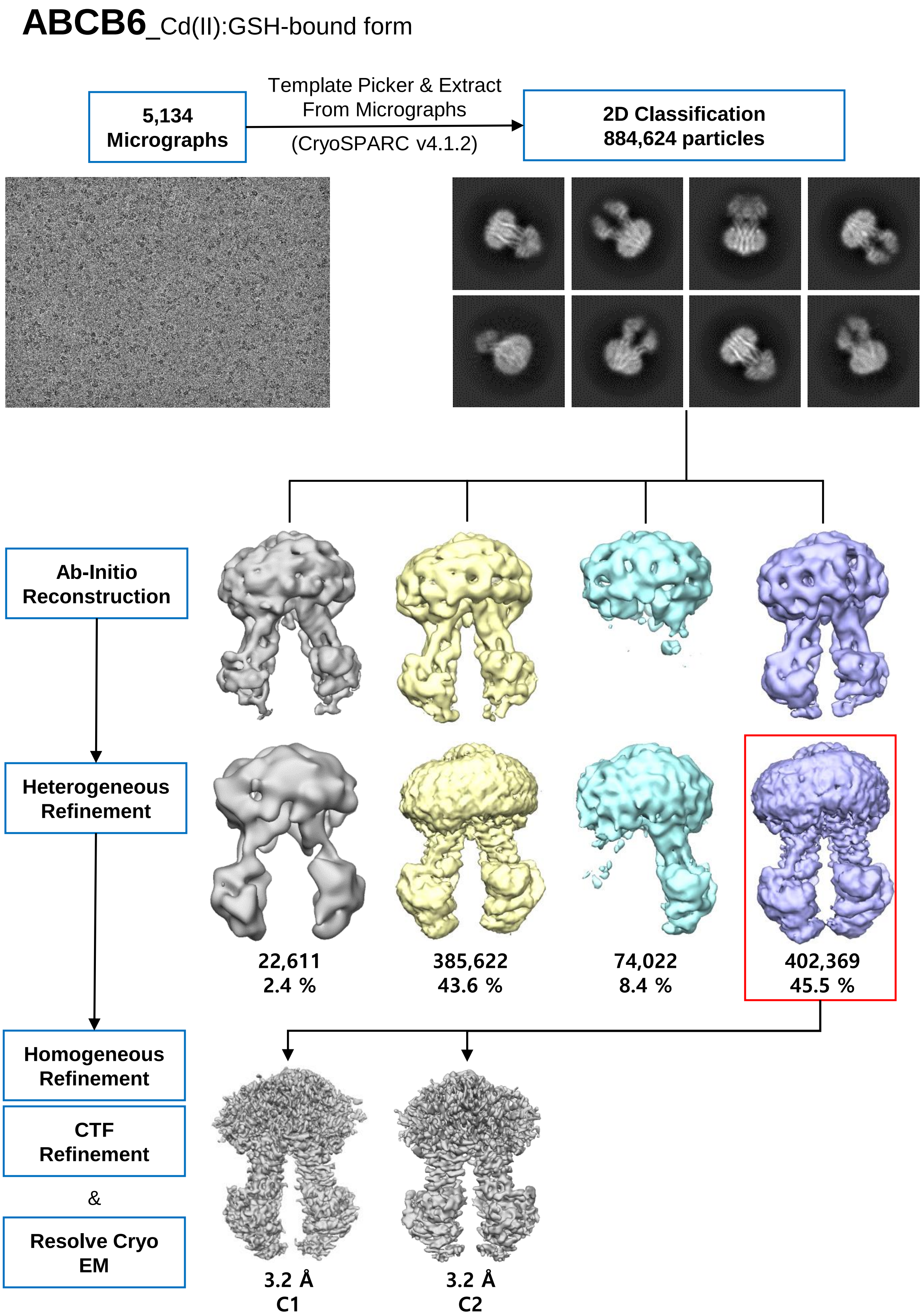

Supplementary Figure 5. Cryo-EM data processing flow chart of Cd(II):GSH-bound hABCB6^{core}. The flow chart for the cryo-EM data processing and structure determination of Cd(II):GSH-bound hABCB6^{core} is presented. Representative micrograph (top, left) and 2D class averages (top, right) are also shown. The 3D heterogeneous refinement subclass subjected to the final 3D reconstruction is marked in a red box.

ABCB6_Cd(II):GSH-bound form

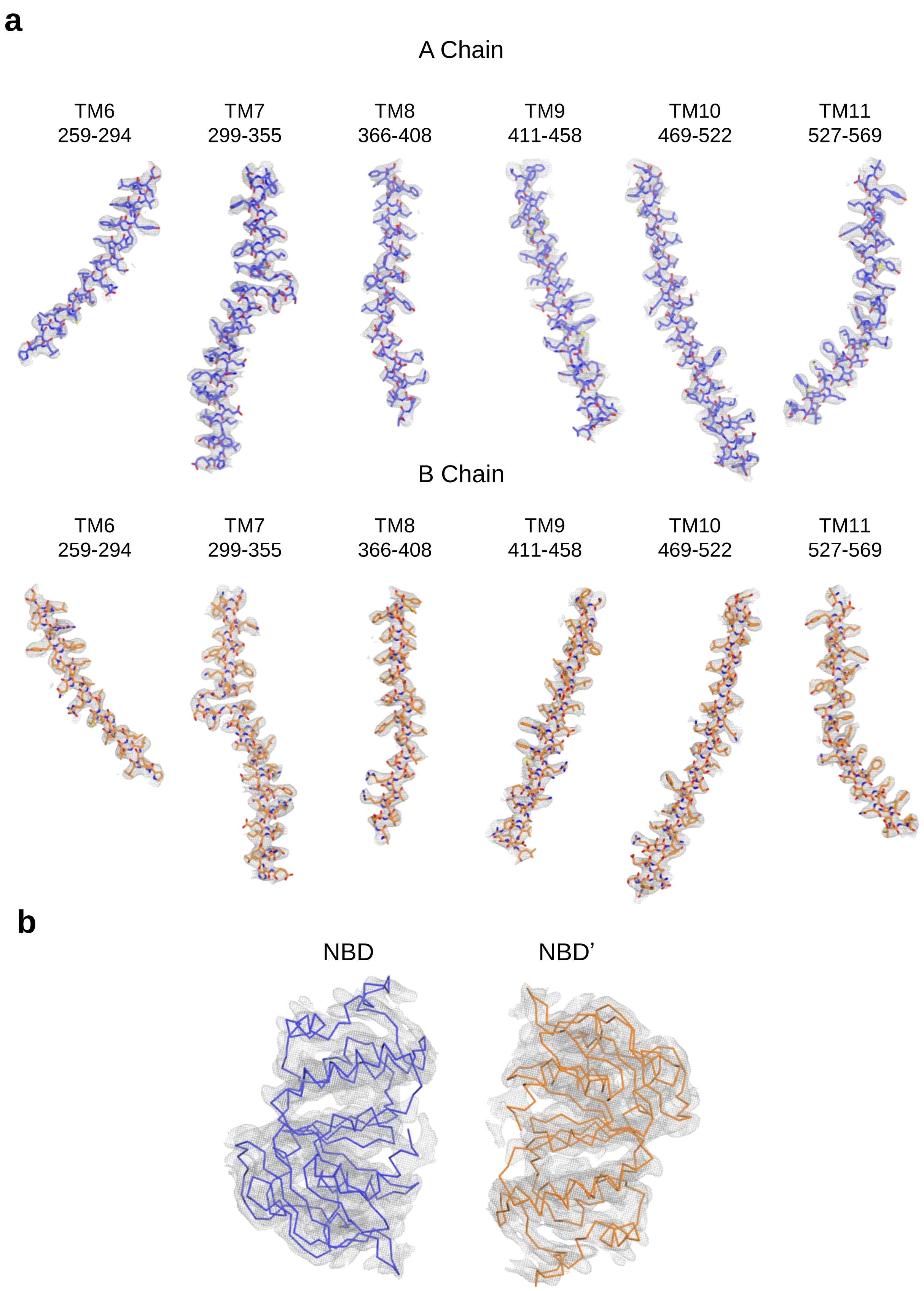

Supplementary Figure 6. Cryo-EM density maps of Cd(II):GSH-bound hABCB6^{core}. (a) Amino acid residues of each TM helix are shown. Density maps (gray mesh) are contoured at the 4 σ level. (b) EM density of the NBD region.

ABCB6_Cd(II):PC2-bound form

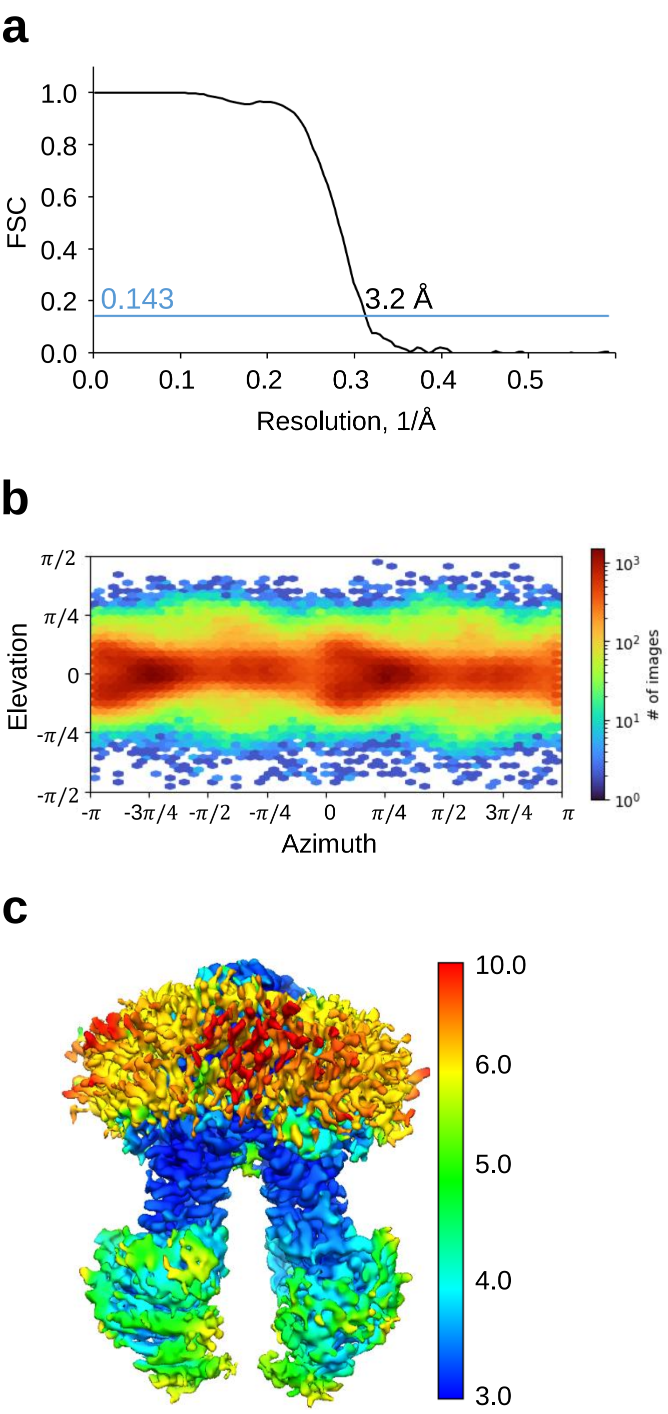

Supplementary Figure 7. Cryo-EM data and model quality analysis of Cd(II):PC2-bound hABCB6^{core}. (a) Gold-standard Fourier Shell correlation (FSC) curve. The resolution estimation (blue line) was calculated based on an FSC cut-off of 0.143. (b) Particle distribution plot for the final 3D reconstruction. (c) Density map colored by local resolution estimation from 3 Å (blue) to 10 Å (red).

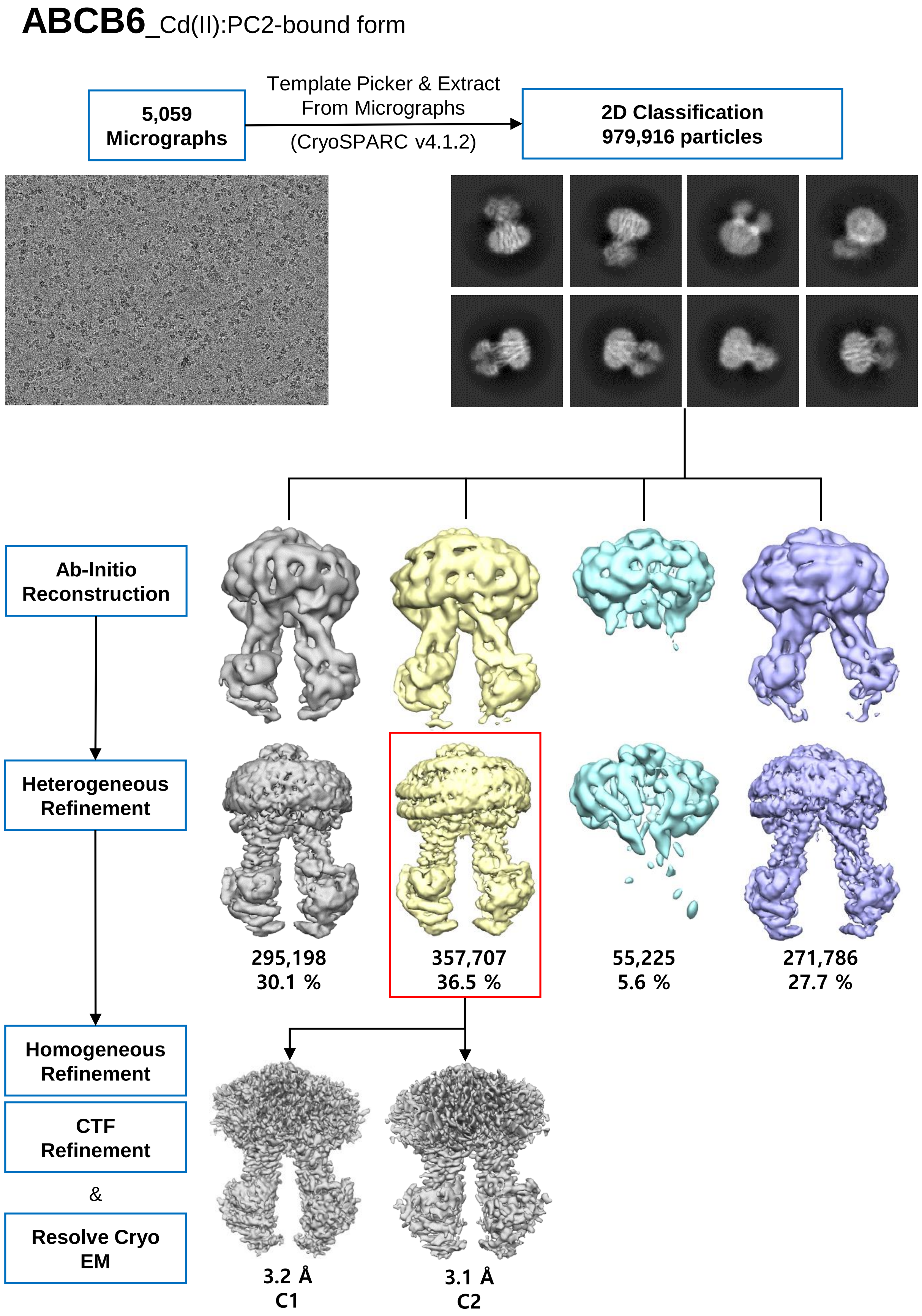

Supplementary Figure 8. Cryo-EM data processing flow chart of Cd(II):PC2-bound hABCB6^{core}. The flow chart for the cryo-EM data processing and structure determination of Cd(II):GSH-bound hABCB6^{core} is presented. Representative micrograph (top, left) and 2D class averages (top, right) are also shown. The 3D heterogeneous refinement subclass subjected to the final 3D reconstruction is marked in a red box.

ABCB6_Cd(II):PC2-bound form

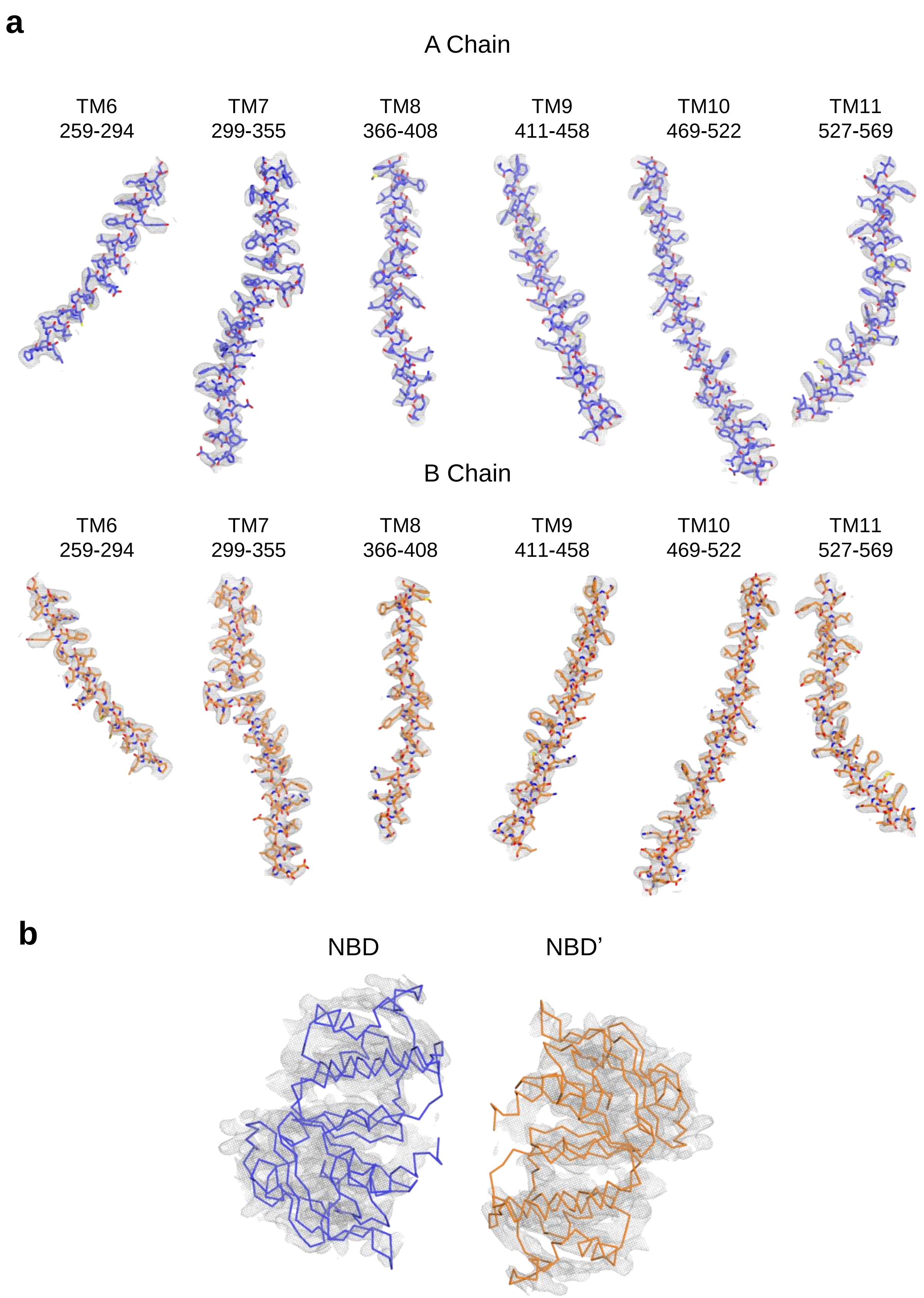

Supplementary Figure 9. Cryo-EM density maps of Cd(II):PC2-bound hABCB6^{core}. (a) Amino acid residues of each TM helix are shown. Density maps (gray mesh) are contoured at the 4 σ level. (b) EM density of the NBD region.

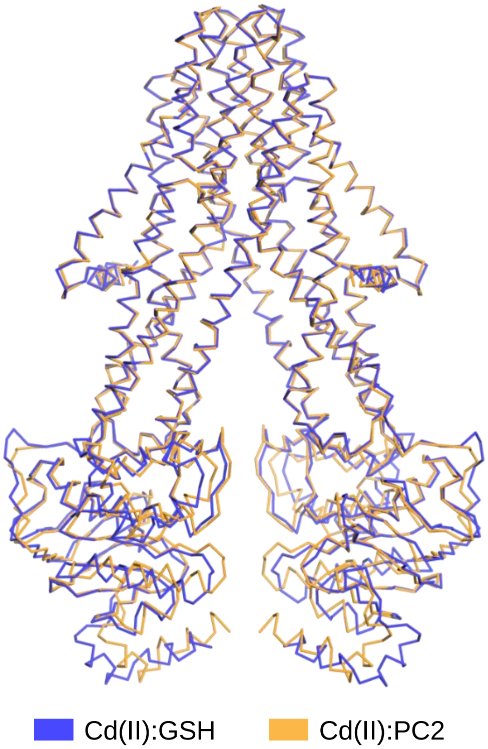

Supplementary Figure 10. Structural comparison of Cd(II)-bound hABCB6^{core} in complex with GSH or PC2. The overall structures of Cd(II):GSH-bound and Cd(II):PC2-bound forms are superimposed, and the two structures are nearly identical (Cα r.m.s.d. = 0.6 Å).

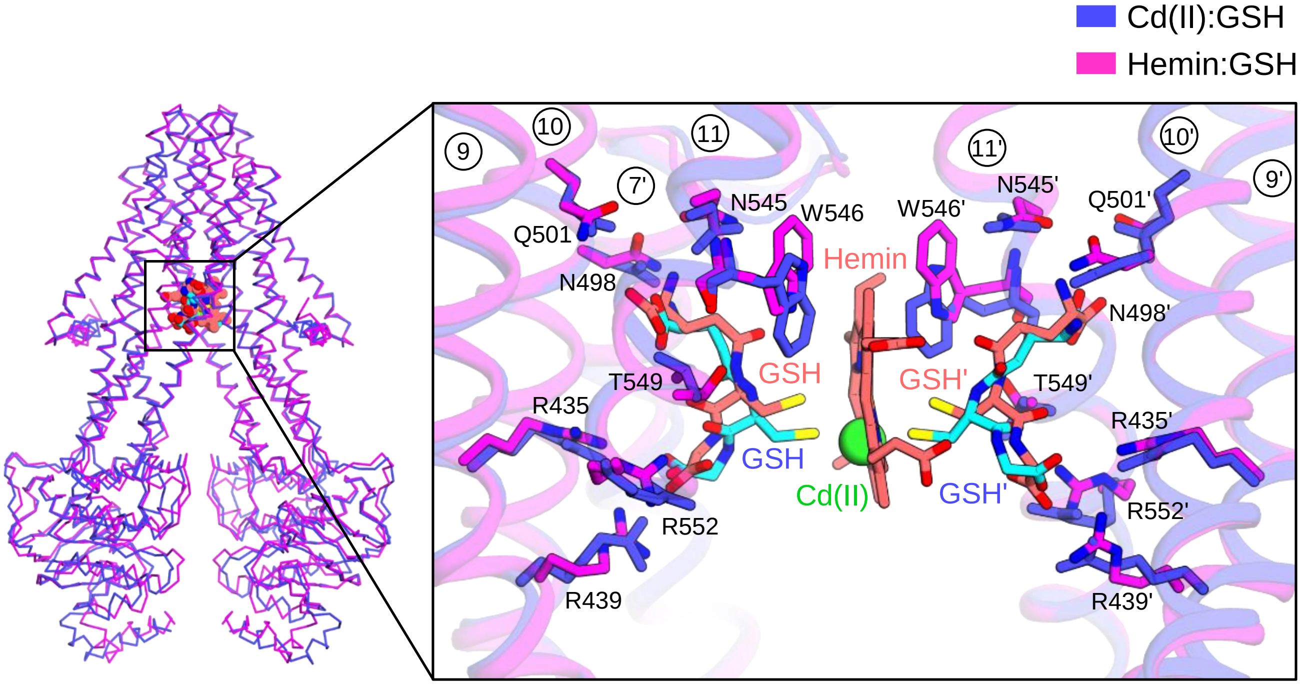

Supplementary Figure 11. Structure comparison of the substrate-binding site of hABCB6^{core}. The hemin:GSH- (PDB ID 7DNZ) and Cd(II):GSH-bound structures align with a Cα r.m.s.d. of 1.3 Å.

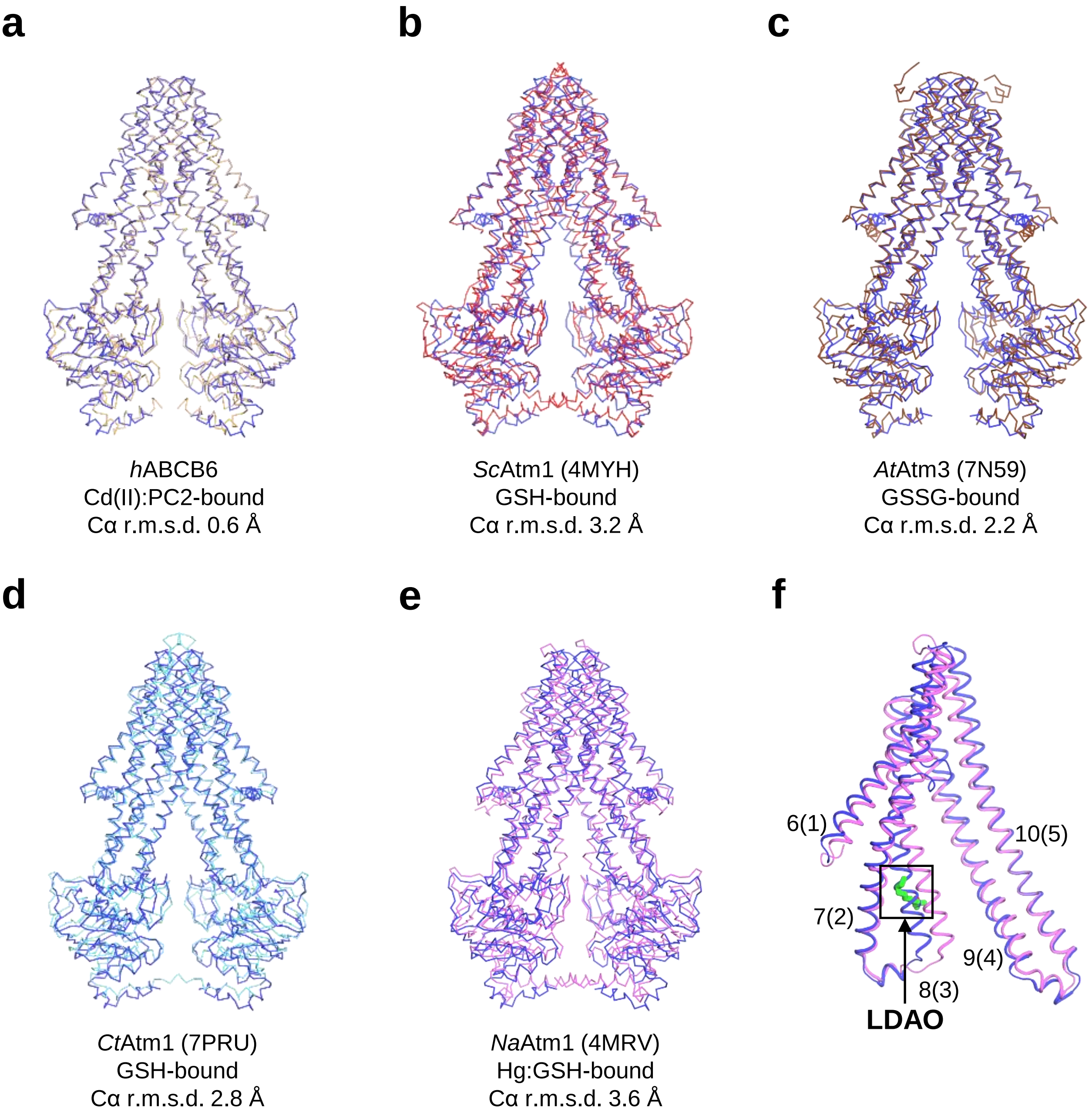

Supplementary Figure 12. Structural comparison of substrate-bound hABCB6^{core} and Atm transporters. (a–e) Superimposition of Cd(II):GSH-bound hABCB6^{core} (blue) with equivalent conformations of Cd(II):PC2-bound hABCB6^{core} (a, orange), Atm1 from *Saccharomyces cerevisiae* (b, red), *Chaetomium thermophilum* (d, cyan), *Novosphingobium aromaticivorans* (e, pink), and Atm3 from *Arabidopsis thaliana* (c, brown). f. Structural comparison of one subunit of Cd(II):GSH-bound hABCB6^{core} and Hg(II):GSH-bound *Na*Atm1. The bound detergent lauryldimethylamine oxide (LDAO) in *Na*Atm1 is depicted as green sticks. The TM helix number in parentheses indicates the corresponding helix in *Na*Atm1.

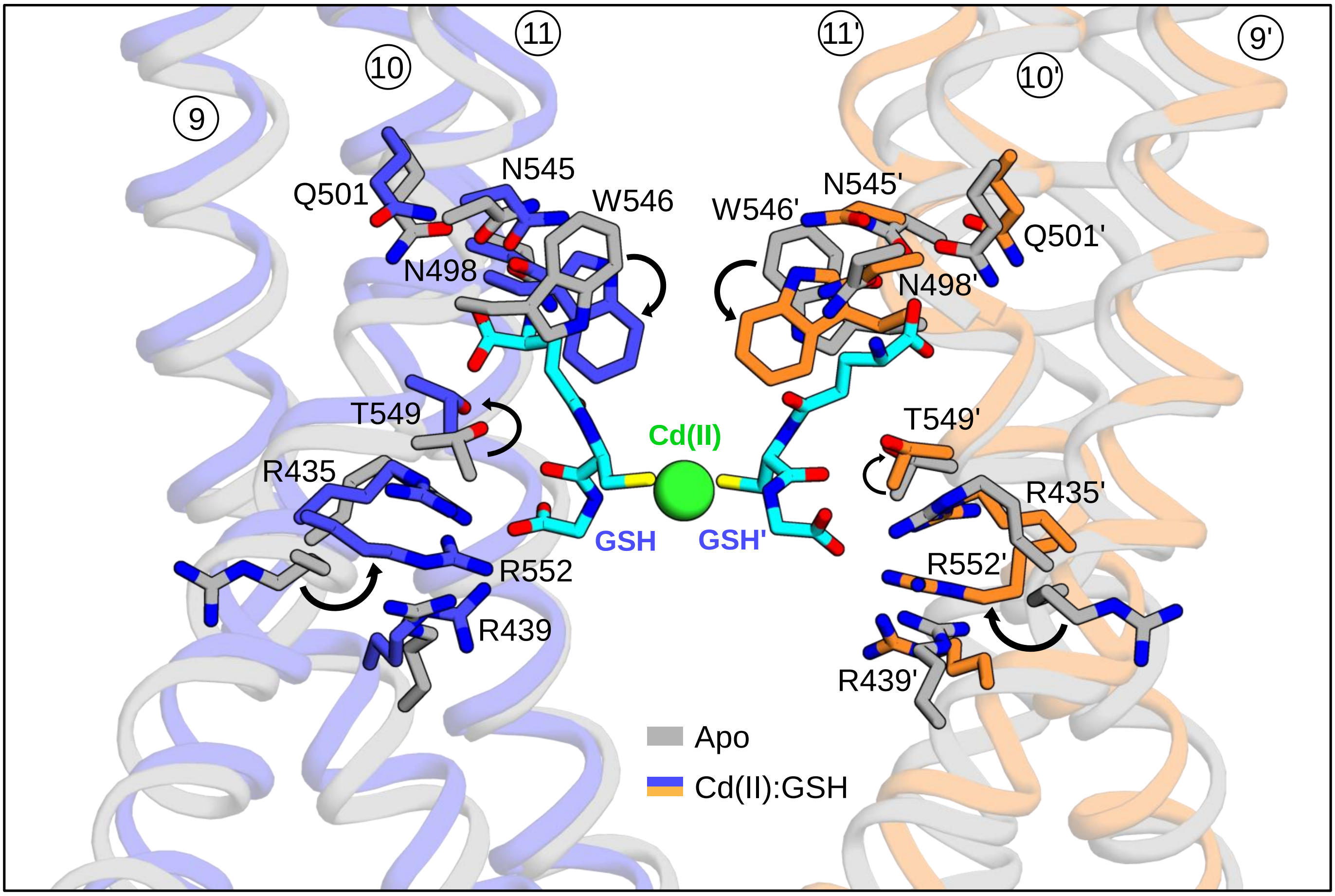

Supplementary Figure 13. Structural comparison of the substrate-binding sites with and without Cd(II):GSH. Structural changes are indicated by black arrows.

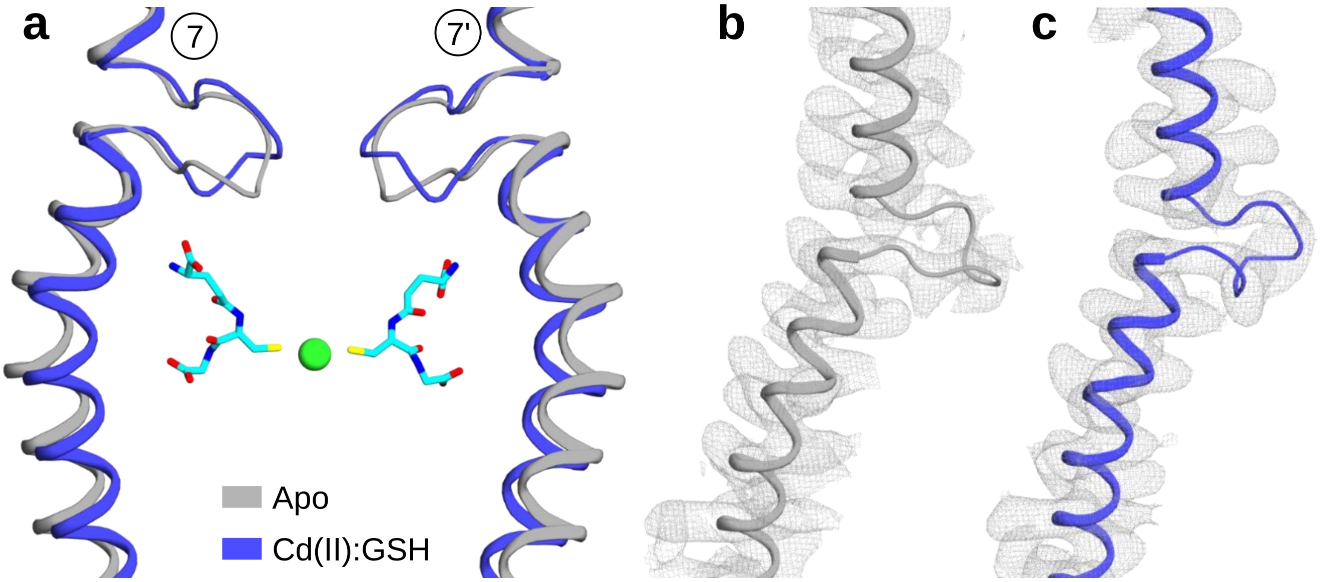

Supplementary Figure 14. Structure changes in the hABCB6^{core} TM 7 loop upon Cd(II):GSH binding. (a) Superimposition of Cd(II):GSH-bound (blue) with apo hABCB6^{core} (gray). (b, c) Density maps (gray mesh) with the TM7 helix of apo hABCB6^{core} (b, gray) and Cd(II):GSH (c, blue). All density maps are contoured at the 3 σ level.

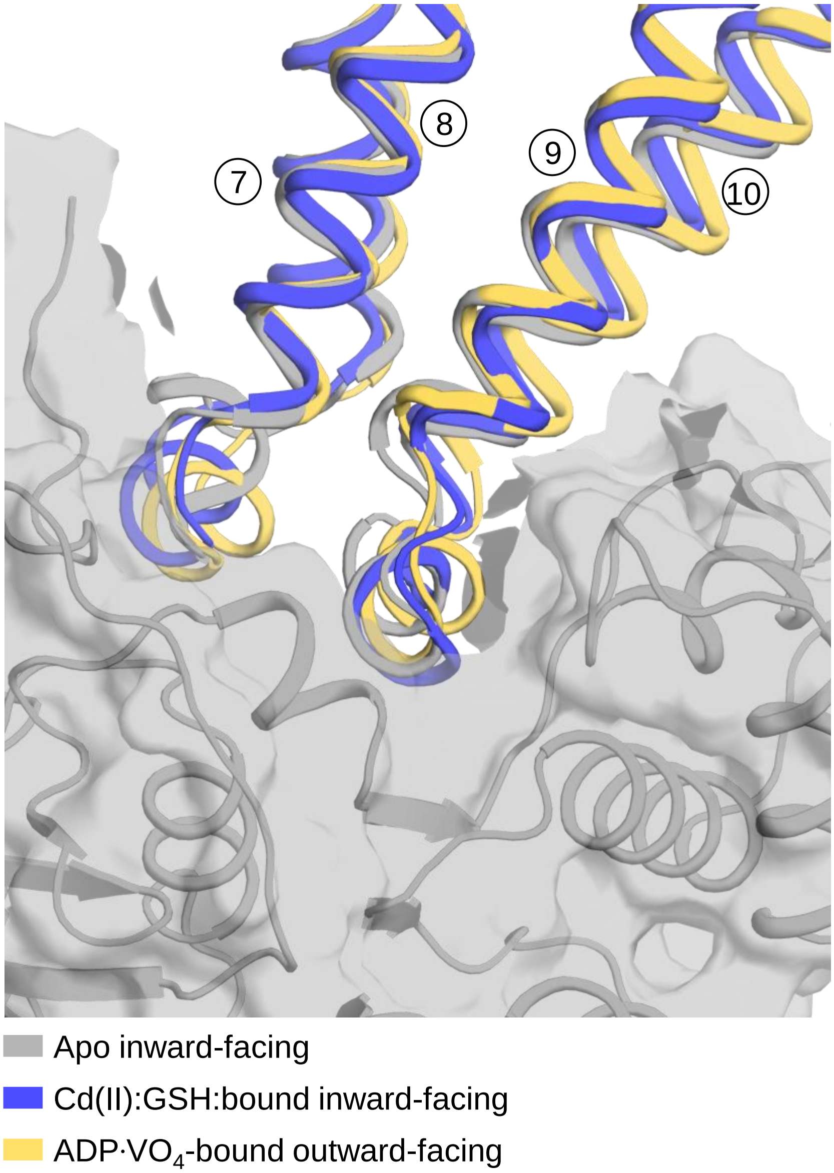

Supplementary Figure 15. The TMD-NBD interface of hABCB6^{core}. The structures of hABCB6^{core} in the apo inward-facing (gray), Cd(II):GSH-bound inward-facing (blue), and ADP·VO_4-bound outward-facing (yellow) states are superimposed with respect to the NBDs.
