# Supplementary material for: Cryo-EM structure of cadmium-bound human ABCB6
Source: Commun Biol. 2024 May 31;7:672. doi: 10.1038/s42003-024-06377-1 (PMC11143254; doi:10.1038/s42003-024-06377-1)
Supplement: Supplementary file 5 — Reporting Summary [file 42003_2024_6377_MOESM5_ESM.pdf]

nature portfolio

Corresponding author(s): Mi Sun Jin

Last updated by author(s): May 6, 2024

Reporting Summary

Nature Portfolio wishes to improve the reproducibility of the work that we publish. This form provides structure for consistency and transparency in reporting. For further information on Nature Portfolio policies, see our Editorial Policies and the Editorial Policy Checklist.

Statistics

For all statistical analyses, confirm that the following items are present in the figure legend, table legend, main text, or Methods section.

n/a	Confirmed	
☐	☒	The exact sample size (n) for each experimental group/condition, given as a discrete number and unit of measurement
☐	☒	A statement on whether measurements were taken from distinct samples or whether the same sample was measured repeatedly
☐	☒	The statistical test(s) used AND whether they are one- or two-sided<br>*Only common tests should be described solely by name; describe more complex techniques in the Methods section.*
☒	☐	A description of all covariates tested
☒	☐	A description of any assumptions or corrections, such as tests of normality and adjustment for multiple comparisons
☐	☒	A full description of the statistical parameters including central tendency (e.g. means) or other basic estimates (e.g. regression coefficient) AND variation (e.g. standard deviation) or associated estimates of uncertainty (e.g. confidence intervals)
☐	☒	For null hypothesis testing, the test statistic (e.g. F, t, r) with confidence intervals, effect sizes, degrees of freedom and P value noted<br>*Give P values as exact values whenever suitable.*
☒	☐	For Bayesian analysis, information on the choice of priors and Markov chain Monte Carlo settings
☒	☐	For hierarchical and complex designs, identification of the appropriate level for tests and full reporting of outcomes
☒	☐	Estimates of effect sizes (e.g. Cohen's d, Pearson's r), indicating how they were calculated

Our web collection on statistics for biologists contains articles on many of the points above.

Software and code

Policy information about availability of computer code

Data collection	All cryo-EM data were collected using a 200kV Talos Arctica, equipped with a K3 BioQuantum direct electron detector and BioQuantum 20 eV energy filter. The micrographs were collected using EPU automated acquisition software v2.8.0.
Data analysis	CryoSPARC v4.1.2<br>- Patch Motion Correction, Patch CTF Estimation, Blob Picker and Template Picker job were done.<br>- Particle extraction, 2D classification, Ab-initio, Heterogeneous 3D refinement and homogeneous 3D refinement were performed.<br>- Global and local CTF refinement, local resolution estimation were processed.<br><br>PHENIX v1.20.1<br>- Resolve Cryo EM were performed.<br><br>Model building<br>- UCSF Chimera v1.16, UCSF ChimeraX v1.16.1, PHENIX v1.20.1 and COOT v0.9.6 were used.<br>- Refined models were validated using MolProbity v4.5.2.<br>- All structure representations in the manuscript were generated using UCSF Chimera v1.16, UCSF ChimeraX v1.16.1, PyMOL v2.4.0 and Prism 10.

For manuscripts utilizing custom algorithms or software that are central to the research but not yet described in published literature, software must be made available to editors and reviewers. We strongly encourage code deposition in a community repository (e.g. GitHub). See the Nature Portfolio guidelines for submitting code & software for further information.

Data

Policy information about availability of data

All manuscripts must include a data availability statement. This statement should provide the following information, where applicable:

- Accession codes, unique identifiers, or web links for publicly available datasets
- A description of any restrictions on data availability
- For clinical datasets or third party data, please ensure that the statement adheres to our policy

We provided public PDB and EMDB accession codes. All of raw data are available from corresponding author upon reasonable request.

Research involving human participants, their data, or biological material

Policy information about studies with human participants or human data. See also policy information about sex, gender (identity/presentation), and sexual orientation and race, ethnicity and racism.

Reporting on sex and gender	N/A
Reporting on race, ethnicity, or other socially relevant groupings	N/A
Population characteristics	N/A
Recruitment	N/A
Ethics oversight	N/A

Note that full information on the approval of the study protocol must also be provided in the manuscript.

Field-specific reporting

Please select the one below that is the best fit for your research. If you are not sure, read the appropriate sections before making your selection.

☒ Life sciences ☐ Behavioural & social sciences ☐ Ecological, evolutionary & environmental sciences

For a reference copy of the document with all sections, see nature.com/documents/nr-reporting-summary-flat.pdf

Life sciences study design

All studies must disclose on these points even when the disclosure is negative.

Sample size	Size exclusion chromatography of the target protein showed a single peak elution at a position corresponding to the molecular weight of 143 kDa, which is expected homodimer size of the target protein.
Data exclusions	Micrographs that showed low CTF resolution or high astigmatism were excluded.
Replication	All replicates were successful.
Randomization	N/A
Blinding	N/A

Reporting for specific materials, systems and methods

We require information from authors about some types of materials, experimental systems and methods used in many studies. Here, indicate whether each material, system or method listed is relevant to your study. If you are not sure if a list item applies to your research, read the appropriate section before selecting a response.

Materials & experimental systems

n/a	Involved in the study
☒	☐ Antibodies
☐	☒ Eukaryotic cell lines
☒	☐ Palaeontology and archaeology
☒	☐ Animals and other organisms
☒	☐ Clinical data
☒	☐ Dual use research of concern
☒	☐ Plants

Methods

n/a	Involved in the study
☒	☐ ChIP-seq
☒	☐ Flow cytometry
☒	☐ MRI-based neuroimaging

Eukaryotic cell lines

Policy information about cell lines and Sex and Gender in Research

Cell line source(s)	Hi5(RRID:CVCL_C190, Expression Systems, LLC), Sf9(RRID:CVCL_0518, Expression Systems, LLC)
Authentication	N/A
Mycoplasma contamination	N/A
Commonly misidentified lines (See ICLAC register)	N/A

Plants

Seed stocks	N/A
Novel plant genotypes	N/A
Authentication	N/A
